# Enhancing UAV object detection with an efficient multi-scale feature fusion framework

**DOI:** 10.1371/journal.pone.0332408

**Published:** 2025-10-08

**Authors:** Delun Lai, Kai Kang, Ke Xu, Xuzhe Ma, Yue Zhang, Fengling Huang, Jishizhan Chen

**Affiliations:** 1 School of Systems and Computing, University of New South Wales, Canberra, Australia; 2 School of Bussiness, University of New South Wales, Sydney, New South Wales, Australia; 3 Faculty of Engineering, University of New South Wales, Sydney, New South Wales, Australia; 4 Faculty of Engineering, University of Sydney, Sydney, New South Wales, Australia; 5 Faculty of Information Technology, Monash University, Melbourne, Victoria, Australia; 6 School of Aeronautics and Astronautics, Shanghai Jiao Tong University, Shanghai, China; 7 UCL Mechanical Engineering, Torrington Place University College London, London, United Kingdom; Purdue University, UNITED STATES OF AMERICA

## Abstract

The rapid advancement of Unmanned Aerial Vehicle (UAV) technology has facilitated dynamic, high-resolution remote sensing, significantly benefiting applications in agriculture, forestry, urban planning, and disaster management. However, detecting small objects in UAV imagery remains challenging due to severe scale variations and environmental complexities. While traditional detection methods and even many advanced YOLO variants achieve reasonable performance, they often either incur high computational costs or fail to preserve the fine-grained features essential for reliably detecting extremely small targets. To overcome these limitations, we propose SRD-YOLOv5, an enhanced version of the lightweight YOLOv5n model, distinguished by its novel multi-scale feature fusion framework. Our approach introduces two innovative modules: the Scale Sequence Feature Fusion Module (SSFF) and the Multi-Scale Feature Extraction Module (MSFE), which collaboratively capture global contextual information and preserve detailed semantic cues that are typically lost in conventional fusion techniques. Furthermore, we incorporate an Extremely Small Target Detection Layer (ESTDL) specifically designed to retain high-resolution features for micro-scale object detection. Additionally, the implementation of a Decoupled Head, which independently processes regression and classification tasks, further optimizes the detection of small targets by reducing task conflicts and improving localization precision. Experimental results demonstrate that SRD-YOLOv5 outperforms existing methods in detecting small targets within UAV remote sensing images. It achieves higher accuracy while maintaining low computational demands, making it suitable for real-time applications in UAV remote sensing.

## Introduction

With the rapid advancement of Unmanned Aerial Vehicle (UAV) technology, remote sensing has achieved dynamic, high-resolution observations of the geographical environment, significantly advancing applications in agriculture, forestry, urban planning, and disaster management [1]. The effectiveness of target detection algorithms in automated remote sensing image analysis is critical to the practical value of UAV imagery. In particular, the ability to accurately detect small targets is crucial, as they often carry essential information but are easily overlooked due to complex backgrounds and limited spatial resolution [2–4].

Despite significant progress in modeling and deep learning [5–9], remote sensing images captured by UAVs still pose challenges stemming from large-scale variations, diverse shooting angles, and complex environmental factors such as lighting and weather [3,4,10]. Traditional object detection methods often struggle with high computational costs, suboptimal adaptability to changing conditions, and insufficient accuracy, particularly when small targets are densely distributed or severely occluded. This necessitates new strategies that can effectively preserve fine-grained feature information, robustly fuse multi-scale features, and maintain real-time performance on resource-constrained UAV platforms.

Recent years have seen remarkable advancements in deep learning-based object detection frameworks, which can be broadly divided into one-stage and two-stage methods [11–25].

Two-stage algorithms typically start by generating candidate boxes, followed by feature extraction and classification using convolutional neural networks, and conclude with target localization refinement through post-processing operations, such as Regional Convolutional Neural Networks (e.g., R-CNN [23], Faster R-CNN [24], and Mask R-CNN [25]). While two-stage algorithms have significantly advanced detection accuracy and target localization, they remain insufficient for satisfying real-time processing requirements.

In contrast, one-stage detectors like YOLO [11–13] integrate classification and localization into a single step, enabling faster inference while maintaining competitive accuracy. Successive YOLO iterations, from YOLOv1 to YOLO11 [14–18,20–22], have introduced anchor-based mechanisms, pyramid network structures, and a variety of architectural optimizations to improve small object detection.

Although these one-stage detectors have proven effective, deploying them in UAV scenarios still faces hurdles [14–18,20–22]. On the one hand, many state-of-the-art YOLO variants involve large backbone networks and complex feature fusion modules, which may not be ideal for real-time processing on UAV platforms with limited onboard computing resources. On the other hand, lightweight models such as YOLOv5n [15] reduce computational overhead but often struggle to detect densely clustered, small-scale targets. Recent methods aimed at overcoming these limitations (e.g. YOLO-LITE [26], YOLO-TLA [27], and LE-YOLO [28]) successfully reduce model size and computational cost. However, they still struggle to balance efficiency with the robust detection of extremely small objects in cluttered environments. Similarly, approaches focusing on small object detection in UAV imagery (e.g., UAV-YOLOv8 [29], HSP-YOLOv8 [4], YOLO-Drone [30], Drone-YOLO [31], and YOLOX_w [32,33]) enhance feature fusion and introduce novel loss functions or attention mechanisms to improve small-scale target detection. Yet, despite these advancements, challenges remain in achieving high detection performance for small objects while maintaining a lightweight architecture suitable for UAV deployment. Balancing the trade-offs among model complexity, computational efficiency, and detection accuracy remains a key research objective, particularly under harsh imaging conditions or when targets appear at extremely small scales.

To address these limitations, this paper proposes SRD-YOLOv5, a multi-scale feature fusion framework that enhances the lightweight YOLOv5n model for more robust small-target detection in UAV remote sensing imagery. Specifically, we aim to: (1) preserve fine-grained details that are crucial for accurate small-object recognition; (2) extract and fuse multi-scale features more effectively; and (3) maintain a compact network architecture suitable for deployment under resource constraints. The key contributions of this work are summarized as follows:

- We propose an enhanced version of the YOLOv5n model, named SRD-YOLOv5, which incorporates a novel multi-scale feature fusion algorithm for UAV remote sensing target detection. By integrating a multi-scale feature fusion mechanism, our approach significantly improves the detection of small targets, particularly excelling in scenarios involving dense small targets within complex backgrounds.
- We developed the Scale Sequence Feature Fusion Module (SSFF) and the Multi-Scale Feature Extraction Module (MSFE). These modules enhance the model’s ability to capture global multi-scale information while effectively preserving semantic details during the feature fusion process.
- Furthermore, we designed an Extremely Small Target Detection Layer (ESTDL) to improve the detection of small targets in high-resolution images. This layer significantly enhances the sensitivity and accuracy of the detection head by refining feature representations, thereby strengthening the model’s ability to locate and identify small targets.
- Finally, we propose a Decoupled Head that adopts a decoupling strategy to separate the processing paths for regression and classification tasks. This design enhances the accuracy of small-target localization, enabling the model to handle different tasks more efficiently and thereby improving overall detection performance.

## Related work

In recent years, with the advancement of object detection, both two-stage and single-stage methods have been proposed [11–25]. While two-stage methods typically offer higher accuracy, single-stage approaches have gained attention for real-time applications—particularly in resource-constrained scenarios such as UAVs [34]. Among single-stage models, the YOLO family achieves an optimal trade-off between speed and accuracy, making it particularly effective for UAV deployments [11–22].

### Object detection for UAV applications

**Two-Stage Methods:** Two-stage detection methods first generate candidate bounding boxes through a Region Proposal Network (RPN), followed by classification and bounding box refinement for each candidate [35]. For example, Faster R-CNN uses an RPN that shares convolutional features to efficiently generate candidate regions. In the second stage, it classifies and predicts object categories and bounding box locations [24]. Mask R-CNN extends Faster R-CNN by adding a mask prediction branch for instance segmentation, while retaining the detection branch with minimal computational overhead [25]. Two-stage methods typically achieve high detection accuracy and robust instance recognition, excelling in complex scenarios. However, their substantial computational overhead and slower inference speed impose significant hardware requirements, limiting real-time applicability on resource-constrained UAV platforms [36]. Despite this limitation, these methods remain valuable for offline analyses where accuracy is prioritized over real-time performance.

**Single-Stage Methods:** Single-stage detectors perform object localization and classification in a single forward pass, without a separate region proposal step. For instance, the Single Shot Detector (SSD) directly regresses bounding boxes and predicts classes on multi-scale feature maps, while the YOLO series divides the image into grids and simultaneously predicts bounding boxes and class probabilities using anchor boxes [35]. These detectors significantly improve detection speed by eliminating sequential RoI cropping and classification steps, making them particularly suitable for real-time UAV applications. However, SSD has demonstrated limited effectiveness in detecting small objects captured by UAVs, performing notably worse than advanced YOLO models and Faster R-CNN [37]. Advanced YOLO variants (e.g., YOLOv3 [13], YOLOv4 [14], YOLOv5 [15]) have effectively integrated multi-scale feature fusion, enhanced backbone architectures, and refined loss functions, significantly improving detection accuracy while maintaining high frame rates. Single-stage detectors, especially the YOLO series, provide an optimal balance between computational efficiency and accuracy for real-time UAV tasks.

**Transformer-Based Methods:** Transformer-based approaches introduce transformer architectures [38] into object detection tasks. For example, DETR formulates detection as a set prediction problem, extracting global context through a Transformer following CNN-based feature extraction to directly output object bounding boxes and class labels, without requiring region proposals or non-maximum suppression (NMS) [39]. The Swin Transformer, a hierarchical visual Transformer, replaces traditional backbones such as ResNet in detection frameworks (e.g., Faster R-CNN, Mask R-CNN) by modeling long-range dependencies through sliding-window self-attention while preserving local receptive fields [40]. DINO further improves upon DETR by incorporating contrastive denoising training and hybrid queries to enhance detection accuracy and accelerate training convergence [41]. Nonetheless, Transformer-based models are characterized by substantial parameter sizes and high computational costs. For instance, Swin-L contains approximately 280 million parameters, and DINO with a ResNet-50 backbone achieves inference speeds of only around 5 FPS [41]. While Transformers excel in capturing global contextual relationships beneficial for UAV imagery with densely distributed small objects and significant scale variations, their computational demands severely constrain real-time deployment on UAV platforms.

Therefore, YOLO-based models remain the preferred choice for UAV object detection. The single-stage architectures of YOLO enable fast inference speeds and real-time performance, satisfying the demanding operational requirements of UAVs [42]. Moreover, advancements in the YOLO series have improved detection accuracy while maintaining model efficiency, further reinforcing their suitability for resource-constrained UAV applications.

### YOLO

YOLOv1 [11], introduced by Joseph Redmon *et al*. in 2016, marked a significant shift from multi-stage pipelines to a single-stage, end-to-end detection framework. By predicting both bounding boxes and class probabilities using a single neural network, YOLOv1 achieved faster inference speeds than earlier approaches such as Fast R-CNN [24] and SSD [43]. However, it struggled with detecting small objects and handling complex scenes involving multiple targets. YOLOv2 [12] introduced anchor boxes, batch normalization, and high-resolution classifiers, enhancing the model’s generalization capability and detection accuracy, particularly for smaller objects. YOLOv3 [13] further built on these improvements by incorporating a multi-scale detection approach that predicts objects at three different scales. It also adopted the deeper Darknet-53 backbone with residual connections, improving feature extraction and gradient flow. These advancements made the model more effective at detecting objects of varying sizes and complexities—a capability particularly beneficial for UAV applications. In 2020, YOLOv4 [14], developed by Alexey Bochkovskiy, introduced major innovations by integrating CSPDarknet53, spatial pyramid pooling (SPP), and path aggregation networks (PAN). These improvements enhanced multi-scale feature extraction, while the adoption of the Mish activation function improved training stability. Released in the same year, YOLOv5 [15] emphasized usability and flexibility by transitioning to the PyTorch framework. It introduced key innovations, including auto-learning of anchor boxes, mosaic data augmentation, and hyperparameter optimization, making it more accessible to developers. YOLOv5 quickly gained popularity in various UAV applications, such as smart farming and real-time surveillance, due to its ease of use and fast inference speeds.

In 2022, YOLOv6 [16], developed by Meituan Vision AI, was designed for industrial applications. It incorporated CSPDarknet as the backbone and enhanced its architecture with a feature pyramid network (FPN) to improve multi-scale detection. YOLOv6 demonstrated superior efficiency and speed, surpassing its predecessors and proving highly effective for UAV-based monitoring systems. YOLOv7 [17] introduced notable advancements in efficiency and scalability. It implemented the Extended Efficient Layer Aggregation Network (E-ELAN), enhancing gradient flow and supporting deeper network designs without compromising learning capacity. Furthermore, the model adopted scalable concatenation-based architecture, making it adaptable to a wide range of hardware platforms, from high-performance servers to embedded devices. In 2023, YOLOv8 [18] introduced a paradigm shift with its anchor-free design, eliminating the reliance on predefined anchor boxes. Instead, it directly predicted object centers, reducing post-processing time and improving accuracy. Additionally, it featured a user-friendly Python interface and online data augmentation, enhancing accessibility for developers.

Released in early 2024, YOLOv9 [20] introduced two core innovations: the Programmable Gradient Information (PGI) framework and the Generalized Efficient Layer Aggregation Network (GELAN). These advancements addressed bottlenecks in information flow and enabled lightweight models to achieve high accuracy, enhancing performance across both deep and shallow architectures. Later in 2024, YOLOv10 [21] further advanced real-time detection by eliminating non-maximum suppression (NMS) through a dual-label assignment strategy. This significantly reduced inference time, making it well-suited for autonomous drones and agricultural monitoring applications.

### YOLO variants for UAV applications

Recent research on object detection in UAV imagery has extensively focused on enhancing the YOLO framework to improve small object detection—a challenging task due to low resolution, complex backgrounds, and overlapping targets. Numerous studies have proposed modifications to YOLOv5, YOLOv8, and YOLOX, emphasizing improvements in accuracy, efficiency, and real-time deployment [4,26–29,31].

To address the computational limitations of UAV platforms, several lightweight versions of YOLO have been developed. YOLO-LITE [26] simplifies the original YOLO framework by reducing the number of convolutional layers and employing depthwise separable convolutions, thereby minimizing memory usage and computational requirements. Despite its lightweight design, it maintains reasonable detection accuracy, making it suitable for real-time surveillance and wildlife tracking on drones with limited processing capabilities. Similarly, YOLO-TLA [27] aims to combine the advantages of tiny models with high-performance architectures. By integrating channel attention mechanisms and employing aggressive down-sampling, it ensures efficiency without compromising detection quality. These enhancements enable the model to achieve high accuracy while maintaining low computational requirements, making it well-suited for UAV applications such as disaster response, traffic monitoring, and autonomous navigation. LE-YOLO [28] builds upon YOLOv8n, focusing on reducing model size and computational overhead while maintaining high detection accuracy. It incorporates a lightweight backbone (LHGNet), efficient neck modules, and a specialized detection head designed for small-object recognition.

Several studies have addressed the challenge of detecting small objects in UAV imagery. Recent modifications to YOLOv5 [2,3] focus on redesigned anchor sizes, attention modules, and the implementation of Complete IoU (CIoU) loss for improved bounding box prediction. Additionally, the introduction of a P2 feature layer helps preserve fine-grained features during detection, enhancing the model’s ability to identify small objects. UAV-YOLOv8 [29] introduces modifications to the YOLOv8 framework to improve small-object detection in complex aerial environments. The model incorporates the Wise-IoU (WIoU) v3 loss function for enhanced localization, BiFormer attention mechanisms for improved feature selection, and a multiscale feature fusion network to strengthen detection across different scales. These enhancements make UAV-YOLOv8 highly effective for UAV-based applications where object size and scene complexity vary significantly. HSP-YOLOv8 [4] focuses on enhancing detection performance for small objects in UAV imagery. The model incorporates a tiny prediction head for high-resolution detection, a Space-to-Depth Convolution (SPD-Conv) module to minimize feature loss during down-sampling, and Soft Non-Maximum Suppression (Soft-NMS) to reduce missed detections. These improvements make HSP-YOLOv8 well-suited for cluttered environments, ensuring greater detection reliability in aerial applications with dense or complex backgrounds.

To address the challenges of varying altitudes, dynamic backgrounds, and overlapping targets in UAV-based detection, YOLO-Drone [30] incorporates multi-scale feature extraction techniques to enhance the detection of objects at different scales. Additionally, the inclusion of an enhanced Region Proposal Network (RPN) reduces false positives, making YOLO-Drone well-suited for applications in urban environments and forests, where objects may be occluded or camouflaged.

Drone-YOLO [31] enhances the YOLOv8 framework by introducing new architectural components to improve small object detection and handle overlapping targets. It incorporates an additional detection head, a sandwich-fusion module, and RepVGG blocks to enhance feature extraction. These modifications make Drone-YOLO particularly effective for UAV tasks in complex environments, such as urban surveillance and environmental monitoring, where small and overlapping objects are common.

YOLOX_w [32] is a modified version of YOLOX-X [33], designed for UAV aerial photography. The model employs image slicing techniques (SAHI) to more effectively detect small objects and integrates ULSAM attention mechanisms to enhance feature learning. Furthermore, it replaces the standard IoU loss with SIoU loss to improve localization accuracy. These modifications enable YOLOX_w to perform well in UAV applications that require precise small-object detection across varying scales.

While these models demonstrate impressive improvements by adding lightweight backbones, extra prediction heads, or enhanced loss functions, they often address scale adaptation and fine-grained feature preservation separately or mainly rely on additional heads and post-processing. To overcome these limitations, we propose SRD-YOLOv5, which differs from existing methods by introducing a dedicated multi-stage feature refinement framework. Unlike LE-YOLO’s [28] backbone optimization or Drone-YOLO’s sandwich-fusion, SRD-YOLOv5 integrates a novel Scale Sequence Feature Fusion (SSFF) and Multi-Scale Feature Extraction (MSFE) to progressively enhance global context and detailed semantics. Moreover, our Extremely Small Target Detection Layer (ESTDL) specifically retains high-resolution cues for micro-scale objects, and a Decoupled Head minimizes task conflict between classification and regression—features that are not jointly addressed in prior works such as HSP-YOLOv8 [4] or Drone-YOLO [31].

By unifying these modules, SRD-YOLOv5 achieves stronger adaptability to severe scale variation while maintaining real-time performance with low computational cost, effectively balancing accuracy and efficiency for UAV-based small object detection in challenging remote sensing scenarios.

## Methods

**Fig 1:**
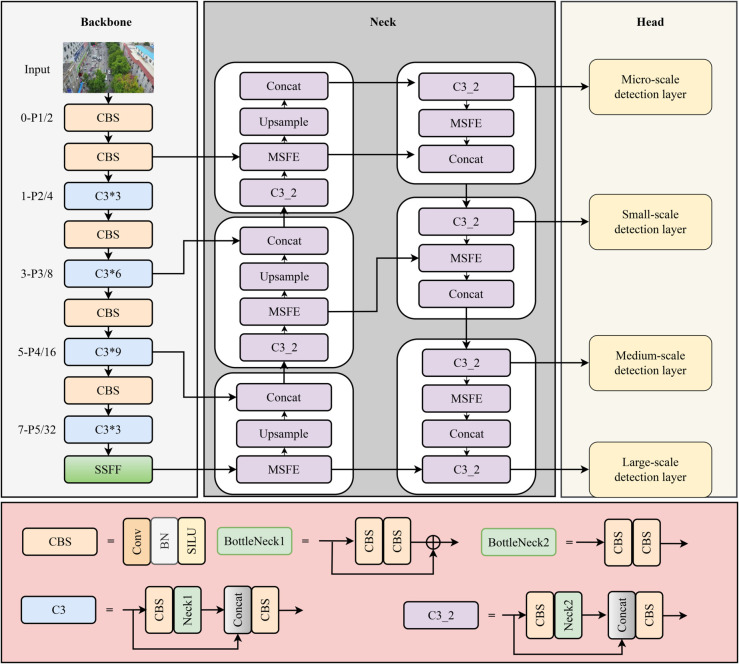
The architecture of SRD-YOLOv5 model.

This section introduces SRD-YOLOv5, a multi-scale feature fusion method for UAV-based remote sensing object detection. The overall framework is illustrated in Fig 1. The backbone adopts YOLOv5n for feature extraction, the neck integrates a Multi-Scale Feature Fusion Layer, and the head incorporates an Extremely Small Target Detection Layer along with a Decoupled Detection Head. These components work together to improve the detection of small targets in remote sensing images. First, the Multi-Scale Feature Fusion Layer is presented, combining a Multi-Scale Feature Extraction Module and a Scale Sequence Feature Fusion Module. This layer enhances the extraction of global multi-scale information in dense small-target environments, preserving semantic features and enriching positional detail. Second, the Extremely Small Target Detection Layer is designed to retain high-resolution detail and refine feature representations, significantly increasing the sensitivity of the detection head to small targets. Finally, a Decoupled Detection Head with two parallel branches for regression and classification further improves the model’s ability to localize small targets accurately.

### Multi-scale feature extraction and fusion layer

In convolutional neural networks, features from different layer scales convey distinct types of information. Low-level features are rich in positional details, while high-level features contain extensive semantic information. YOLOv5n employs an FPN+PAN pyramid structure to fuse feature maps extracted by the backbone, enhancing semantic information transmission. However, frequent up-sampling and down-sampling operations within the FPN+PAN pyramid can lead to the loss of small target details in dense UAV remote sensing images, resulting in suboptimal small target detection. Furthermore, the PAN’s bottom-up fusion of feature maps at the same scale fails to effectively integrate rich feature information from various scales, thereby reducing model performance.

To address these limitations, this paper introduces the Scale Sequence Feature Fusion Module (SSFF) and the Multi-Scale Feature Extraction Module (MSFE). These modules are designed to enhance the network’s ability to extract global multi-scale information for densely distributed small targets while effectively fusing feature maps across different scales. This integration not only preserves the semantic information of targets but also retains rich positional details.

#### Multi-scale feature extraction module.

**Fig 2:**
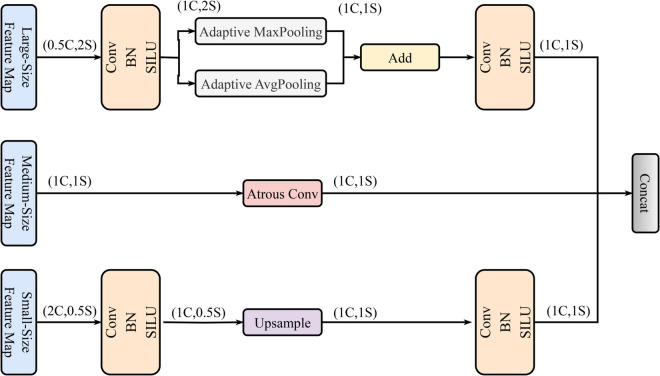
The structure of MSFE module.

Traditional FPN fusion mechanisms typically up-sample small-scale feature maps and add them to the previous layer, often overlooking the rich details in large-scale feature layers. To address this limitation, we designed the MSFE module. As shown in Fig 2, the MSFE module captures and combines detailed multi-scale feature information by separately processing large-, medium-, and small-scale features.

The MSFE module consists of three parallel branches:

(1) **Upper branch**: Processes large-scale feature maps *F*_*L*_.

(2) **Middle branch**: Processes medium-scale feature maps *F*_*M*_.

(3) **Lower branch**: Processes small-scale feature maps *F*_*S*_.

The operations in each branch are formalized as follows:

**Upper branch**: A 1×1 convolution adjusts the channel dimensions of large-scale feature maps, followed by adaptive max-pooling and average-pooling to down-sample the features.

$$F_{L}^{\prime }={\text{Conv}}_{1\;\times \;1}(F_{L}),$$ (1)

$$F_{L}^{\prime \prime }=\text{Concat}(\text{MaxPool}(F_{L}^{\prime }),\text{AvgPool}(F_{L}^{\prime })).$$ (2)

**Middle branch**: For medium-scale feature maps, the channel dimensions are adjusted using:

$$F_{M}^{\prime }={\text{Conv}}_{1\;\times \;1}(F_{M}).$$ (3)

**Lower Branch**: For small-scale feature maps, we adjust the channels and up-sample:

$$F_{S}^{\prime }={\text{Conv}}_{1\;\times \;1}(F_{S}),$$ (4)

$$F_{S}^{\prime \prime }=\text{Upsample}(F_{S}^{\prime }).$$ (5)

After processing each branch, we concatenate the features:

$$F_{\text{MSFE}}=\text{Concat}(F_{L}^{\prime \prime },F_{M}^{\prime },F_{S}^{\prime \prime }).$$ (6)

This concatenated feature map $F_{\text{MSFE}}$ retains comprehensive multi-scale information, enhancing the detection accuracy for small targets in UAV remote sensing imagery.

#### Scale sequence feature fusion module.

**Fig 3:**
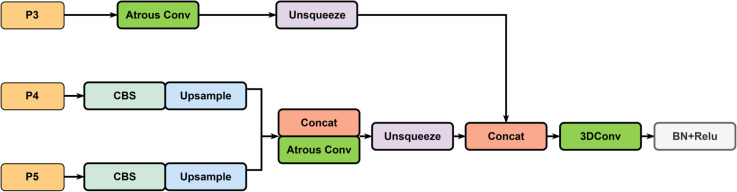
The structure of SSFF module.

Since the high-resolution feature levels *P*2 and *P*3 contain the majority of key information for small object detection, we use the *P*3 level as an example to design the SSFF (as shown in Fig 3). The input to the SSFF consists of three components: *P*3, *P*4, and *P*5, representing feature maps with sizes of 80×80, 40×40, and 20×20, respectively.

First, the feature map from the *P*3 level undergoes atrous convolution to increase the receptive field and enhance the network’s ability to detect small objects:

$$P3^{\prime }=\text{AConv}(P3)$$ (7)

where AConv denotes atrous convolution.

Next, the feature maps from the *P*4 and *P*5 levels are processed using 1×1 convolutions to match the number of channels to *P*3, followed by up-sampling to resize the feature maps to 80×80:

$$P4^{\prime }=\text{Upsample}\left({\text{Conv}}_{1\;\times \;1}(P4)\right)$$ (8)

$$P5^{\prime }=\text{Upsample}\left({\text{Conv}}_{1\;\times \;1}(P5)\right)$$ (9)

Subsequently, the feature maps from the $P3^{\prime }$, $P4^{\prime }$, and $P5^{\prime }$ levels are concatenated:

$$F_{\text{concat}}=\text{Concat}(P3^{\prime },P4^{\prime },P5^{\prime })$$ (10)

Atrous convolution is applied to the concatenated features to avoid information loss while maintaining resolution.

$$F_{\text{concat}}^{{\;\;}^{\prime }}=\text{AConv}(F_{\text{concat}};d^{\prime })$$ (11)

Finally, the processed feature maps are passed through a 3D convolution layer to extract scale sequence features:

$$F_{\text{SSFF}}=\text{Conv3D}(F_{\text{concat}}^{{\;\;}^{\prime }})$$ (12)

By extracting scale sequence features through 3D convolution and leveraging the invariance of scale features, the method effectively combines the high-level semantic information from deep feature maps at different scales with the detailed information from shallow feature maps, thereby strengthening the fusion of multi-scale information. This process ensures an effective integration of high-level semantic information with detailed positional features, significantly enhancing the detection of small targets.

### Extremely small target detection layer

**Fig 4:**
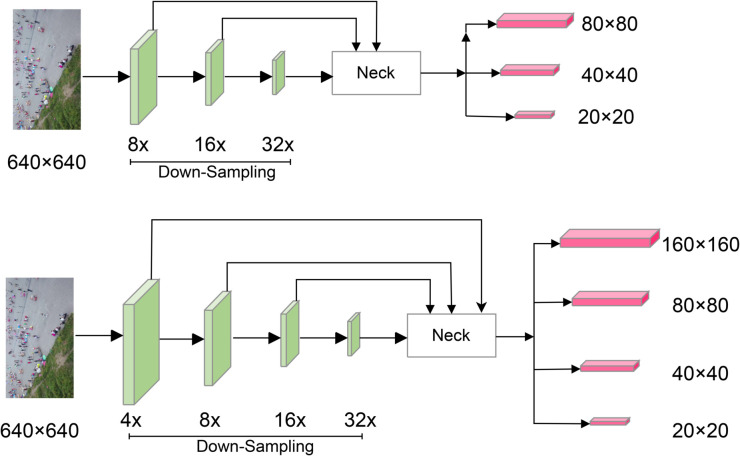
The structure of extremely small target detection module.

Fig 4 compares the original YOLOv5n detection network structure with the enhanced architecture incorporating the Extremely Small Target Detection Layer (ESTDL). In YOLOv5n, when the input image size is 640×640, the backbone feature extraction network performs 8×, 16×, and 32× down-sampling to extract feature information. The neck component then fuses features from the backbone using the FPN+PAN feature fusion network. Finally, the head outputs three feature maps of different sizes: 80×80, 40×40, and 20×20, corresponding to the detection of small, medium, and large objects, respectively. While this structure performs well on datasets like COCO, where target category sizes are balanced, it faces challenges with UAV remote sensing images, where small targets predominate. In these images, some targets are smaller than 8×8 pixels. Even the small target detection head, with a size of 80×80, struggles to make accurate predictions.

To address this, we introduce a new 4× down-sampling layer, *P*2 (Extremely Small Target Detection Layer, ESTDL), with a feature map size of 160×160, specifically designed to detect extremely small targets in remote sensing images. This high-resolution layer preserves rich positional information and refined feature details, effectively enhancing the sensitivity of the detection head and improving detection accuracy (Table 1).

**Table 1:** Detailed configurations and deployment considerations of the proposed MSFE, SSFF, and ESTDL modules.

Module	Branch/Layer	Operation	Kernel Size / Dilation	Activation	I/O Size (Example)
MSFE	Upper	1×1 Conv	1×1, dil=1	ReLU	256×128×128 → 128×128×128
	Upper	Adaptive MaxPool	–	–	128×128×128 → 128×64×64
	Upper	Adaptive AvgPool	–	–	128×128×128 → 128×64×64
	Middle	1×1 Conv	1×1, dil=1	ReLU	256×64×64 → 128×64×64
	Lower	1×1 Conv + Upsample	1×1, dil=1	ReLU	256×32×32 → 128×64×64
SSFF	P3	Atrous Conv	3×3, dil=2	ReLU	256×80×80 → 128×80×80
	P4	1×1 Conv + Upsample	1×1, dil=1	ReLU	512×40×40 → 128×80×80
	P5	1×1 Conv + Upsample	1×1, dil=1	ReLU	1024×20×20 → 128×80×80
	Fusion	Atrous Conv	3×3, dil=2	ReLU	384×80×80 → 128×80×80
	Extractor	3D Conv	3×3×3	ReLU	(1×128×80×80) → (1×128×80×80)
ESTDL	Backbone	3×3 Conv	3×3, dil=1	ReLU	128×320×320 → 128×160×160
	Head	1×1 Conv	1×1, dil=1	Sigmoid	128×160×160 → output

### Decoupled head layer

The YOLOv5 algorithm employs a coupled detection head to perform object category classification and bounding box regression on feature maps from the FPN+PAN feature fusion network. However, in object detection tasks, researchers have observed that classification and regression focus on different aspects, and using the same feature layer for both tasks can reduce model performance. Specifically, classification tasks emphasizes the object’s salient features, while regression tasks prioritize the object’s positional information.

**Fig 5:**
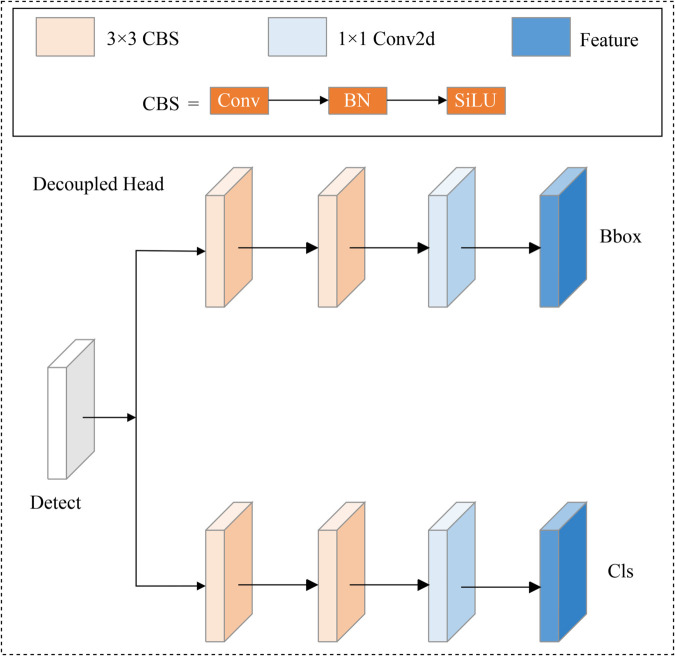
The structure of decoupled head module.

In UAV remote sensing images, where targets are small, backgrounds are complex, and objects are difficult to locate, the coupled detection head can reduce target detection accuracy. Inspired by YOLOX, we designed a Decoupled Detection Head with two parallel branches to handle regression and classification tasks separately, thereby improving the model’s sensitivity to small object localization. Fig 5 illustrates the structure of the Decoupled Detection Head module. Formally, for each feature map $F_{\text{in}}$ from the neck network, the Decoupled Detection Head functions as follows:

**Regression Branch**:

$$F_{\text{reg}}={\text{Conv}}_{3\;\times \;3}^{2}(F_{\text{in}})$$ (13)

$${𝐭}_{\text{reg}}={\text{Conv}}_{1\;\times \;1}(F_{\text{reg}})$$ (14)

**Classification Branch**:

$$F_{\text{cls}}={\text{Conv}}_{3\;\times \;3}^{2}(F_{\text{in}})$$ (15)

$${𝐭}_{\text{cls}}={\text{Conv}}_{1\;\times \;1}(F_{\text{cls}})$$ (16)

Here, $\text{Conv}{3\;\times \;3}^{2}$ denotes two consecutive 3×3 convolutional layers, and 𝐭reg, ${𝐭}_{\text{cls}}$ denote the outputs for regression (bounding box coordinates) and classification (object classes), respectively.

The design of the Decoupled Head effectively balances the focus of both tasks, enhancing the model’s performance by allowing each branch to specialize in its respective task.

### Loss function

The SRD-YOLOv5 model utilizes both regression and classification loss functions to predict object categories and perform bounding box regression. Specifically, it employs Complete Intersection over Union (CIoU) Loss and Distribution Focal Loss (DFL) for regression, and Varifocal Loss (VFL) for classification. The detailed formulations of these loss functions are as follows:

$$L_{CloU}=1-IoU+\frac{\rho^{2}\left(b,b^{gt}\right)}{c^{2}}+\frac{\nu^{2}}{\left(1-IoU\right)+\nu },$$ (17)

where IoU represents the Intersection over Union between the predicted bounding box and the ground truth bounding box. *b* and *b*^*gt*^ denote the centers of the predicted and ground truth boxes, respectively. $\rho (b,b^{gt})$ is the Euclidean distance between the centers of the predicted and ground truth boxes. *c* represents the diagonal length of the minimum enclosing box containing both the predicted and ground truth boxes. ν measures the consistency of the aspect ratio between the predicted and ground truth boxes:

$$\nu =\frac{4}{\pi^{2}}{\left(\arctan\frac{w^{gt}}{h^{gt}}-\arctan\frac{w}{h}\right)}^{2}.$$ (18)

$$L_{DFL}=-\left(\left(y_{i+1}-y\right)\log\left(\frac{y_{i+1}-y}{y_{i+1}-y_{i}}\right)+\left(y-y_{i}\right)\log\left(\frac{y-y_{i}}{y_{i+1}-y_{i}}\right)\right),$$ (19)

where *y* represents the integral result, and *y*_*i*_ and *y*_*i* + 1_ are the results at the interval endpoints.

$$L_{VFL(p,q)}=\left\{ \begin{array}{cc} -q\left(q\log(p)+(1-q)\log(1-p)\right), & q>0\\ -\alpha p^{\gamma }\log(1-p), & q=0 \end{array}\right.,$$ (20)

where *p* represents the IACS-predicted value for target confidence, *q* is the classification condition, *α* is a scaling factor, and *γ* is an adjustable ratio factor.

Combining the above components, the total loss function for training the model is:

$$L_{\text{total}}=\lambda_{\text{cls}}\sum_{i}L_{\text{VFL}}(p_{i},q_{i})+\lambda_{\text{reg}}\sum_{i}\left(L_{\text{CIoU}}(b_{i},b_{i}^{\text{gt}})+\sum_{k}L_{\text{DFL}}(b_{i,k},b_{i,k}^{\text{gt}})\right),$$ (21)

where $\lambda_{\text{cls}}$ and $\lambda_{\text{reg}}$ are weighting coefficients.

## Experiments

### Dataset

In the experimental stage, we utilized two public UAV datasets. The VisDrone2019 dataset (All VisDrone2019 files are available from the https://github.com/VisDrone/VisDrone-Dataset.) [44] is a large-scale dataset specifically designed for UAV vision tasks, collected by drones across 14 different cities in China. It includes a variety of weather conditions, shooting angles, and lighting changes, presenting significant challenges for object detection. This dataset includes 8,629 high-resolution images, divided into 6,471 for training, 548 for validation, and 1,610 for testing. It is annotated with 342,391 target instances across 10 categories: pedestrian, people, bicycle, car, van, truck, tricycle, awning-tricycle, bus, and motor.

The RSOD dataset (All RSOD files are available from the https://github.com/RSIA-LIESMARS-WHU/RSOD-Dataset-.) [45], designed for object detection in remote sensing images, includes four typical categories: airplanes, playgrounds, overpasses, and oil drums. It offers a variety of lighting conditions and contrasts, with challenges such as shadows, occlusions, and deformations complicating detection tasks. After excluding some unlabeled images from the original dataset, a total of 936 images were obtained and divided into training, validation, and test sets in an 8:1:1 ratio, with 748 training images, 94 validation images, and 94 test images.

The NWPU VHR-10 dataset [46] is a publicly available remote sensing object detection benchmark containing ten representative categories: airplane, ship, storage tank, baseball diamond, tennis court, basketball court, ground track field, harbor, bridge, and vehicle. The images were cropped from Google Earth and the Vaihingen dataset, then manually annotated by domain experts to ensure high-quality ground truth labels. The dataset provides a total of 800 very high-resolution (VHR) remote sensing images, covering diverse geographic scenes and complex backgrounds. For this study, the images were split into training, validation, and test sets following an 8:1:1 ratio, resulting in 640 training images, 80 validation images, and 80 test images.

### Evaluation metrics

To evaluate the performance of the proposed object detection method, we employed the following metrics: Precision, Recall, Average Precision (AP), and Mean Average Precision (mAP). Precision is defined as the ratio of correctly predicted positive samples (true positives) to all predicted positive samples (true positives and false positives). Recall is the ratio of correctly predicted positive samples to all actual positive samples (true positives and false negatives).

$$\text{Precision}=\frac{TP}{TP+FP}$$ (22)

$$\text{Recall}=\frac{TP}{TP+FN}$$ (23)

where TP denotes the number of correctly predicted remote sensing objects, FP refers the number of incorrectly predicted remote sensing objects, and FN indicates the number of remote sensing objects that were missed.

AP represents the area under the Precision–Recall curve across different threshold settings. It can be interpreted as the model’s average performance over all operating points, providing a comprehensive assessment for individual categories.

$$AP=\int_{0}^{1}P(r)\;dr$$ (24)

mAP represents the average of the APs across all categories and is commonly used for multi-category object detection tasks.

$$mAP=\frac{1}{n}\sum_{i=1}^{n}AP_{i}$$ (25)

GFLOPs (Giga Floating Point Operations per Second): Measures the total number of floating point operations required by the model to process a single image, expressed in billions of operations. It reflects the computational complexity. FPS (Frames Per Second): Represents the number of images that can be processed by the model per second during inference, indicating the inference speed. APS (Average Precision for Small objects), APM (Average Precision for Medium objects), and APL (Average Precision for Large objects): These metrics follow the COCO evaluation protocol and represent the average precision calculated for different object scales. They are defined as:

$$APS=\frac{1}{T}\sum_{t=1}^{T}AP(IoU_{thr}^{\left(t\right)},\text{area}<32^{2})$$ (26)

### Implementation details

All experiments in this study were conducted in a consistent software and hardware environment, specifically: Ubuntu 20.04, an NVIDIA RTX 3070 GPU with 8 GB of VRAM, and the PyTorch 1.12.1 framework with CUDA 11.3 and cuDNN 8.2. To ensure reproducibility, random seeds (111, 222, 333, 444, 555, 666) were explicitly set for all experiments. Model initialization parameters, including weights, were kept consistent across all runs. Evaluation metrics (e.g., Precision, Recall, and mAP) are reported as the mean of values obtained from at least five independent runs, ensuring the reliability and reproducibility of the results.

In this work, we applied widely used data augmentation techniques that are standard in drone-based object detection benchmarks. Specifically, the training images were augmented using random horizontal flipping, random scaling, random cropping, and photometric distortions (including brightness, contrast, and saturation adjustments). These augmentations help increase the diversity of small objects under various backgrounds and scales, which is critical for robust detection performance. In addition, transfer learning was employed by initializing the model backbone with weights pretrained on the COCO dataset, which is a common practice in UAV target detection to provide strong general-purpose feature representations. This pretraining ensures better convergence and performance, especially when training datasets are limited or contain challenging small-scale objects.

During training, the initial learning rate (LR) was set to 0.01, selected after testing candidate values of 0.001, 0.005, 0.01, and 0.02. Over the first three epochs, a linear warm-up gradually increased the LR from 0 to 0.01, preventing instability caused by an excessively large step size at the outset. To further suppress overfitting and ensure stable convergence, the LR was maintained at 0.01 from epochs 1 to 200, after which a cosine annealing strategy was applied until epoch 300, smoothly decaying the LR to 0.0001. Training was performed using stochastic gradient descent (SGD) with a momentum parameter of 0.935 and a weight decay of 0.0005. The batch size was fixed at 16, and training was conducted for a total of 300 epochs. For data preprocessing, all input images were uniformly resized to 640×640 pixels to ensure consistency in data dimensions.

Taking the VisDrone2019 dataset as an example, training for 300 epochs on a single NVIDIA RTX 3070 GPU required approximately 5.0 hours for YOLOv5n, 7.5 hours for YOLOv5s, and about 6.5 hours for the proposed SRD-YOLOv5. Despite incorporating multi-scale feature extraction and a high-resolution detection layer, the increase in training time for SRD-YOLOv5 relative to YOLOv5n remained modest and acceptable, while yielding significant improvements in detection accuracy—particularly for small-scale and densely distributed targets.

## Experiment results and analysis

### Comparison with state-of-the-art on VisDrone2019 dataset

To evaluate the performance of the proposed SRD-YOLOv5 model for UAV remote sensing object detection, we conducted comprehensive experiments on the VisDrone2019 dataset and compared the results against several state-of-the-art detection methods. Table 2 presents the detection accuracies (mAP@0.5 and AP for individual categories) of various models, including DetNet59 [47], DMNet [48], RefineDet [49], EfficientViT [51], YOLOv3-LITE [50], DBAI-Det [47], YOLOv3-tiny [13], YOLOv5n [15], YOLOv5s [15], YOLOv7-T [17], YOLOv8n [18], YOLOv9t [20], YOLOv10-N [21], and YOLO11-N [52].

**Table 2:** Comparison of target detection accuracy with various state-of-the-art models on the VisDrone2019 dataset. Results are reported as mean ± 95% confidence interval.

Methods	mAP@0.5	Pedestrian	People	Bicycle	Car	Van	Truck	Tricyle	Awning Tricyle	Bus	Motor
DetNet [47]	15.3	15.3	4.1	3.1	36.1	17.3	20.9	13.5	10.5	26.0	10.9
DMNet [48]	30.3	28.5	20.4	15.9	56.8	37.9	30.1	22.6	14.0	47.1	29.2
RefineDet [49]	14.9	14.9	3.7	2.02	30.1	16.3	18.1	9.0	10.3	21.9	8.38
YOLOv3-LITE [50]	28.5	34.5	23.4	7.9	70.8	31.3	21.9	15.3	6.2	40.9	32.7
DBAI-Det [47]	28.0	36.7	12.8	14.7	47.4	38.0	41.4	23.4	16.9	31.9	16.6
EfficientViT [51]	31.9	33.7	26.0	7.6	74.8	37.3	27.8	20.6	10.1	45.6	35.7
YOLOv3-tiny [13]	16.8	19.8	19.7	3.44	51.9	14.8	10.4	8.96	3.79	15.6	19.3
YOLOv5n [15]	28.8	31.7	25.3	6.45	67.6	23.3	18.8	12.0	8.8	32.6	31.0
YOLOv5s [15]	33.4	40.9	33.3	10.9	73.8	36.0	27.7	17.8	11.2	42.3	39.9
YOLOv7-T [17]	37.2	40.7	37.7	12.3	77.2	38.3	31.8	24.2	12.7	50.3	46.5
YOLOv8n [18]	33.7	35.0	27.6	8.48	76.0	39.4	28.7	21.4	12.5	49.6	38.0
YOLOv9t [20]	36.7	37.2	30.5	10.0	77.7	42.6	34.1	24.9	14.9	54.0	41.0
YOLOv10-N [21]	34.7	36.4	29.0	10.1	77.0	38.9	31.3	22.5	12.4	50.7	39.1
YOLO11-N [52]	34.7	37.1	28.6	9.7	76.5	40.3	31.8	23.2	12.3	48.2	39.5
SRD-YOLOv5	35.9±0.3	42.2	34.7	10.6	80.0	40.0	28.6	23.0	12.3	45.4	42.3

The results indicate that SRD-YOLOv5 not only raises the overall detection performance (with an mAP@0.5 of 35.9%) but also offers significant improvements in detecting small-scale objects—a common challenge in UAV imagery. The model’s robust performance across various categories, along with its advantages over recent YOLO variants, validates its state-of-the-art capability for UAV remote sensing object detection tasks.

Detection performance for small targets is critical in UAV applications. In the Pedestrian category, SRD-YOLOv5 achieves an AP of 42.2%, compared to 31.7% for YOLOv5n—an improvement of 10.5%. Similarly, for the People category, the AP increases from 25.3% (YOLOv5n) to 34.7% (SRD-YOLOv5), a gain of 9.4%. For the Bicycle category, SRD-YOLOv5 attains 10.6% AP, compared to 6.45% with YOLOv5n, marking an improvement of 4.15%. These results suggest that the model’s architecture—particularly its multi-scale feature fusion and specialized detection layers—is highly effective for identifying small and densely distributed objects. While excelling in small object detection, SRD-YOLOv5 also maintains strong performance for larger objects. The AP for the Car category reaches 80.0%, while the Van and Motor categories achieve 40.0% and 42.3%, respectively. This indicates that the model not only improves detection of small objects but also retains high accuracy for larger targets, ensuring balanced performance across different object sizes.

When compared to the latest variants such as YOLOv8n, YOLOv9t, YOLOv10-N, and YOLO11-N, SRD-YOLOv5 consistently demonstrates competitive or superior performance. YOLOv10-N and YOLOv11-N, while showing respectable results with mAPs around 34.7%, still fall short in key categories (e.g., Pedestrian and People) compared to SRD-YOLOv5. This suggests that the improvements introduced in SRD-YOLOv5—such as the Extremely Small Target Detection Layer and the Decoupled Detection Head—play a critical role in boosting performance.

### Complexity and efficiency analysis

Table 3 presents the performance of various YOLO-based models and the proposed SRD-YOLOv5, evaluated in terms of both detection effectiveness and computational complexity.

**Table 3:** Comparison between SRD-YOLOv5 and YOLO series models on the VisDrone2019 dataset. Results are reported as mean ± 95% confidence interval. Statistical significance compared to SRD-YOLOv5 is tested by paired t-test (: *p*<0.05, *: *p*<0.01, ns: not significant).

Models	Precision (%)	Recall (%)	mAP@0.5 (%)	mAP@0.5:0.95 (%)	FPS	Parameters (M)	GFLOPs
YOLOv3-tiny [13]	30.4	19.3	16.8	-	46	8.68	12.9
YOLOv5n [15]	36.4	27.7	25.8	-	98	1.77	4.2
TPH-YOLOv5 [53]	-	-	35.4	-	-	8.36	18.7
YOLOv5s [15]	44.4${\;\;}^{\text{ns}}$	34.6${\;\;}^{\text{ns}}$	33.4^*^	14.5	45	7.05	16.5
YOLOv8n [18]	44.5${\;\;}^{\text{ns}}$	33.6^*^	33.7^*^	-	95	3.00	8.2
YOLOv10-N [21]	44.9${\;\;}^{\text{ns}}$	34.0${\;\;}^{\text{ns}}$	34.7${\;\;}^{\text{ns}}$	-	88	2.30	6.7
YOLO11-N [52]	45.2${\;\;}^{\text{ns}}$	34.2${\;\;}^{\text{ns}}$	34.7${\;\;}^{\text{ns}}$	-	90	2.60	6.5
Drone-YOLO (nano) [31]	-	-	31.0	17.5	-	3.05	-
Drone-YOLO (tiny) [31]	-	-	33.7	19.1	-	5.35	-
Drone-YOLO (small) [31]	-	-	35.6	20.4	-	10.90	-
YOLO-PoolFormer [54]	35.2	24.0	21.5	-	-	39.26	114.2
YOLO-HV [55]	48.0^*^	38.8^*^	38.1^*^	19.90	-	38.54	111.9
SRD-YOLOv5	45.6	35.8	35.9±0.3	20.20±0.2	102	4.18	12.5

In terms of effectiveness, we assess four critical metrics: Precision, Recall, mAP0.5, and mAP0.5:0.95. SRD-YOLOv5 consistently outperforms lightweight models such as YOLOv3-tiny, YOLOv5n, YOLOv8n, YOLOv10-N, and YOLO11-N, achieving higher values in both mAP0.5 and mAP0.5:0.95. Although certain heavier models, such as YOLO-HV and Drone-YOLO (small), show marginally higher mAP0.5 and mAP0.5:0.95 scores, these gains come at the cost of significantly increased model size and computation.

Regarding complexity, we consider three aspects: (1) the number of parameters (M), which reflects memory footprint and deployability; (2) GFLOPs, which indicate the computational cost per forward pass; and (3) FPS, which measures real-time inference capability. Notably, SRD-YOLOv5 maintains a relatively lightweight configuration with only 4.18M parameters and 12.5 GFLOPs, while achieving 102 FPS, which is comparable to the highest-speed models (e.g., YOLOv5n). Compared with small and nano variants like YOLOv5n and YOLOv8n, SRD-YOLOv5 slightly increases resource usage but delivers substantial improvements in both mAP0.5 and mAP0.5:0.95, particularly for challenging small-object detection tasks.

In contrast, larger models such as YOLO-PoolFormer and YOLO-HV deliver modest improvements in detection performance. For example, YOLO-HV achieves slightly higher Precision and mAP0.5, but its computational cost exceeds 111 GFLOPs—more than nine times that of SRD-YOLOv5. Such high complexity not only demands greater memory and energy but also limits deployment on resource-constrained UAV platforms.

Overall, SRD-YOLOv5 achieves an effective balance between detection accuracy and computational efficiency. Its integration of multi-scale feature fusion and a dedicated detection branch for extremely small targets contributes to improved Precision, Recall, mAP0.5, and mAP0.5:0.95, while keeping the model compact and fast. This balance makes SRD-YOLOv5 well-suited for UAV-based remote sensing tasks where both high detection performance and real-time processing are required.

### Comparison with state-of-the-art on RSOD dataset and NWPU VHR-10 dataset

To validate the robustness and generalizability of the proposed SRD-YOLOv5 model, a series of experiments were carried out on the RSOD remote sensing object detection dataset. As summarized in Table 4, SRD-YOLOv5 consistently delivers improvements in Precision, mAP@0.5, and mAP@0.5:0.95, while maintaining a high inference speed suitable for real-time applications.

**Table 4:** Comparison experiments with various classic models on RSOD dataset.

Methods	Precision (%)	mAP@0.5 (%)	mAP@0.5:0.95(%)	FPS
Faster R-CNN [24]	-	88.7	-	21
SSD [43]	-	87.9	-	27
YOLOv5n [15]	83.9	90.4	81.5	135
YOLOv3-SPP [56]	90.9	95.0	83.6	30
YOLOv3-tiny [13]	89.8	93.4	82.1	56
YOLOv6 [16]	85.3	90.5	80.7	99
YOLOv8 [18]	90.3	89.7	80.2	76
LAR-YOLOv8 [10]	91.5	94.3	83.1	56
SRD-YOLOv5	90.6	94.6±0.4	83.4±0.4	117

In detail, SRD-YOLOv5 achieves a Precision increase of 6.7% (from 83.9% to 90.6%), an mAP@0.5 improvement of 4.2% (from 90.4% to 94.6%), and a gain of 1.9% in mAP@0.5:0.95 (from 81.5% to 83.4%) compared with YOLOv5n, while sustaining an inference speed of 117 FPS, which meets the requirements for real-time deployment.

When evaluated against representative two-stage detectors such as Faster R-CNN and SSD, SRD-YOLOv5 achieves higher mAP@0.5 values by 5.9% and 6.7%, respectively, and significantly improves the inference speed, reaching 117 FPS, in contrast to the 21 FPS and 27 FPS achieved by the two-stage models.

Compared with YOLOv3-tiny, SRD-YOLOv5 demonstrates an increase of 0.8% in Precision, an enhancement of 1.2% in mAP@0.5 (from 93.4% to 94.6%), and an improvement of 1.3% in mAP@0.5:0.95 (from 82.1% to 83.4%), while maintaining more than twice the inference speed (117 FPS compared to 56 FPS). Furthermore, relative to YOLOv6, SRD-YOLOv5 achieves an mAP@0.5 improvement of 4.1% and a gain of 2.7% in mAP@0.5:0.95 (from 80.7% to 83.4%), along with an 18 FPS increase in processing speed.

To further verify the generalization capability and practical effectiveness of SRD-YOLOv5 in remote sensing scenarios, additional experiments were conducted on the NWPU VHR-10 dataset. As shown in Table 5, SRD-YOLOv5 demonstrates competitive advantages over conventional CNN-based methods and state-of-the-art single-stage YOLO detectors.

**Table 5:** Comparison experiments with various classic models on NWPU VHR-10 dataset.

Methods	Precision (%)	mAP@0.5 (%)	mAP@0.5:0.95(%)	FPS
DMNet [57]	75.0	65.1	50.5	-
DSHNet [58]	76.2	66.3	55.1	-
CDMNet [59]	77.3	67.4	52.3	-
UFMP-Net [60]	80.4	70.9	55.7	-
YOLOv5m [15]	81.0	71.3.	57.2	147
YOLOv8m [18]	81.4	71.8±0.2	60.1±0.1	168
SRD-YOLOv5	81.6	74.4±0.3	60.5±0.2	109

For example, compared to representative CNN-based approaches such as DMNet, DSHNet, CDMNet, and UFMP-Net, SRD-YOLOv5 achieves consistent improvements in Precision and mAP metrics. Specifically, SRD-YOLOv5 attains a Precision of 81.6%, exceeding UFMP-Net’s 80.4%, and an mAP@0.5 of 74.4%, surpassing UFMP-Net’s 70.9%. These results illustrate the model’s enhanced ability to detect diverse small targets in complex remote sensing scenes.

Furthermore, relative to mainstream single-stage detectors including YOLOv5m and YOLOv8m, SRD-YOLOv5 attains slightly higher Precision—81.6% compared to 81.0% and 81.4%, respectively—and improved mAP@0.5 of 74.4%, outperforming their 71.3% and 71.8%. Despite these performance gains, SRD-YOLOv5 maintains a real-time inference speed of 109 FPS, confirming its suitability for UAV- and satellite-based remote sensing applications.

These results collectively highlight that the proposed SRD-YOLOv5 model offers superior detection performance and strong generalization capabilities across remote sensing object detection datasets.

### Ablation studies

To validate the effectiveness of the SRD-YOLOv5 model’s modules for small object detection in UAV remote sensing imagery, we conducted detailed ablation experiments on the VisDrone2019 dataset. The results, summarized in Table 6, show the performance impact when each module is added individually to the YOLOv5n baseline.

**Table 6:** Performance comparison of YOLOv5n with various modifications.

Models	Precision (%)	Recall (%)	mAP@0.5 (%)	mAP0.5:0.95(%)	GFLOPs
YOLOv5n	36.4	27.7	25.8	13.1	4.2
+P2 detection	40.5	29.8	30.3	17.2	6.9
+Decoupled Head	43.0	31.2	31.7	18.1	7.1
+SSFF	41.9	30.7	31.2	17.1	2.8
+MSFE	42.5	31.1	31.4	17.8	5.1
+ESTDL	45.6	35.8	35.9	20.2	12.5

To provide a fair baseline comparison for extremely small object detection, we first introduce a P2 detection layer, which is a conventional shallow detection head commonly used to enhance low-level feature resolution. Adding the P2 layer alone increases the mAP@0.5 from 25.8% to 30.3% and mAP@0.5:0.95 from 13.1% to 17.2%, with Precision and Recall also rising to 40.5% and 29.8%, respectively. This demonstrates the benefit of including finer-resolution features for small objects, although the P2 head is not our proposed contribution.

Next, incorporating the Decoupled Head into YOLOv5n improves Precision, Recall, mAP@0.5, and mAP@0.5:0.95 from 36.4%, 27.7%, 25.8%, and 13.1% to 43.0%, 31.2%, 31.7%, and 18.1%, respectively. This shows that decoupling the regression and classification tasks effectively mitigates task conflict, enhancing both localization and classification precision. The added computational cost remains moderate (GFLOPs increase from 4.2 to 7.1).

Adding the SSFF module alone improves mAP@0.5 to 31.2% and mAP@0.5:0.95 to 17.1%, while Precision and Recall reach 41.9% and 30.7%, respectively. Notably, SSFF achieves this gain with a lower GFLOPs (2.8) than the baseline, indicating that the selective scale-sequence fusion effectively enhances multi-scale context representation with minimal computational burden.

Similarly, integrating the MSFE module alone lifts mAP@0.5 to 31.4% and mAP@0.5:0.95 to 17.8%, with Precision and Recall increasing to 42.5% and 31.1%, respectively. This demonstrates that MSFE enriches global contextual information and preserves fine-grained semantic cues across scales while keeping the GFLOPs moderate (5.1).

Finally, adding the ESTDL achieves the most significant performance improvement among all modules, raising Precision to 45.6%, Recall to 35.8%, mAP@0.5 to 35.9%, and mAP@0.5:0.95 to 20.2%. This confirms that ESTDL effectively retains high-resolution feature details critical for detecting extremely small objects in UAV scenes, with an expected increase in GFLOPs to 12.5 for the final configuration.

Together, these results demonstrate that each proposed module contributes meaningfully to the overall performance improvements of SRD-YOLOv5 while maintaining an efficient computational profile suitable for UAV-based deployment.

Table 7 summarizes the performance impact of different initial learning rates (LRs) tested during hyperparameter tuning. Specifically, candidate LR values of 0.001, 0.002, 0.005, 0.01, 0.02, and 0.05 were evaluated. As shown, an initial LR of 0.01 achieved the highest mAP, with 35.9% at mAP@0.5 and 20.2% at mAP@0.5:0.95, outperforming other tested settings. This confirms that setting the initial LR to 0.01 provides a good balance between stable convergence and optimal detection performance.

**Table 7:** Effect of initial learning rate (LR) on mAP.

Value	mAP@0.5 (%)	mAP0.5:0.95(%)
LR=0.001	35.4	19.1
LR=0.002	33.8	18.2
LR=0.005	34.7	18.8
LR=0.01	35.9	20.2
LR=0.02	34.8	18.5
LR=0.05	35.2	19.6

### Performance on small-scale object detection

To verify the effectiveness of the proposed SSFF, MSFE, and ESTDL modules for small-scale object detection, we conducted a series of ablation experiments comparing the YOLOv5n baseline with its enhanced variants that integrate each module individually. The results, summarized in Table 8, report the COCO metrics for average precision on small (APS), medium (APM), and large (APL) objects, as well as inference speed (FPS).

**Table 8:** Performance comparison of YOLOv5n and its enhanced versions incorporating the SSFF, MSFE, and ESTDL modules across object sizes.

	APS (%)	APM (%)	APL (%)	FPS
YOLOv5n	6.3	17.9	24.2	98
YOLOv5n+SSFF	6.5 (↑ 4.1%)	18.1 (↑ 1.2%)	24.8 (↑ 2.5%)	99(↑ 1s)
YOLOv5n+MSFE	6.7 (↑ 5.2%)	18.3 (↑ 2.4%)	25.1 (↑ 3.6%)	100(↑ 2s)
YOLOv5n+ESTDL	8.8 (↑39.7%)	21.4 (↑19.6%)	26.4 (↑9.1%)	102 (↑ 4s)

The inclusion of the SSFF module results in relative improvements of 4.1%, 1.2%, and 2.5% in APS, APM, and APL, respectively, along with a slight increase in inference speed from 98 to 99 FPS. Incorporating the MSFE module yields relative gains of 5.2% in APS, 2.4% in APM, and 3.6% in APL, while maintaining real-time processing at 100 FPS. These results indicate that both SSFF and MSFE effectively enhance feature fusion and multi-scale representation, which are particularly beneficial for detecting small and medium-sized objects.

Furthermore, integrating the ESTDL module leads to substantial improvements of 39.7% in APS, 19.6% in APM, and 9.1% in APL, while increasing the inference speed to 102 FPS. These results demonstrate that ESTDL significantly boosts the detection accuracy of small-scale targets in remote sensing imagery without sacrificing real-time performance.

### Visualization

**Fig 6:**
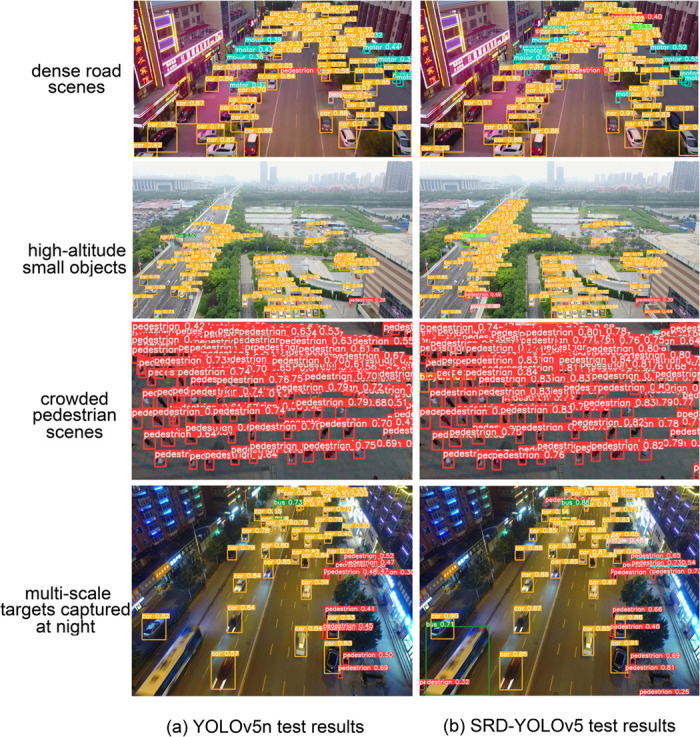
Comparison of detection effects of YOLOv5n (left), and SRD-YOLOv5 (right) in dense road scenes, high-altitude small objects, crowded 522 pedestrian scenes, and multi-scale targets captured at night.

To demonstrate the effectiveness of our proposed method, we selected several challenging images from the VisDrone2019 test set, including scenes with dense road traffic, small objects at high altitudes, crowded pedestrian areas, and multi-scale targets captured during nighttime. Comparative visualization results are presented in Fig 6, where the left images (YOLOv5n) depict baseline performance, and the right images illustrate the superior performance of our method. It can be observed that our method significantly improves detection accuracy, especially for small or densely distributed targets, accurately identifying targets missed or incorrectly classified by the baseline model.

In urban environments, SRD-YOLOv5 demonstrates superior detection capabilities, effectively identifying complex targets in challenging scenarios such as dense road traffic. Its superior performance is evidenced by accurate recognition of densely packed targets, overcoming limitations observed in YOLOv5n. Specifically, SRD-YOLOv5 benefits from the Extremely Small Target Detection Layer (ESTDL), which enhances fine-grained detail preservation, enabling more reliable detection of distant or extremely small targets, compared to YOLOv5n. Furthermore, in scenes characterized by overlapping or densely clustered pedestrians, the decoupled head structure of SRD-YOLOv5 reduces misdetections by separately optimizing classification and localization tasks, yielding clearer distinctions between closely positioned targets. Additionally, in challenging nighttime scenarios with multi-scale targets, the model exhibits robust performance against adverse lighting conditions due to its effective integration of multi-scale feature fusion and enhanced feature extraction modules. Consequently, SRD-YOLOv5 maintains high detection accuracy and demonstrates enhanced robustness under suboptimal lighting conditions.

Overall, SRD-YOLOv5 exhibits strong robustness against interference from complex backgrounds, dense object distributions, and limited target information. As a result, it significantly improves detection accuracy, effectively reducing both false positives and missed detections. These advantages highlight the model’s capability to markedly enhance object detection performance in UAV-based remote sensing applications, especially in challenging real-world conditions.

## Disscssion

To facilitate real-time detection on UAVs, we ensured that SRD-YOLOv5 remains lightweight (approximately 8.4 MB with 4.18M parameters). This compact size is compatible with embedded platforms such as the NVIDIA Jetson Nano and Xavier NX. By converting the model to ONNX and optimizing it via TensorRT, we typically achieve around 30 FPS at a 640×640 resolution on a Jetson device, making the system well-suited for time-critical tasks such as aerial surveillance and target tracking. Additionally, the typical 10–15 W power envelope of these edge devices aligns with UAV battery constraints, enabling multi-rotor drones to perform onboard object detection without significantly impacting flight time.

## Limitations

In practical applications, although SRD-YOLOv5 has achieved significant improvements in multi-scale feature fusion and extremely small target detection, several limitations and challenges remain. Methodologically, the approach is still constrained by the inherent biases and labeling precision of the training datasets, which may lead to generalization issues when deployed in more complex or imbalanced real-world scenarios. In terms of performance, the trade-off between detection accuracy and real-time processing must be carefully managed—particularly under extreme weather conditions, low-light environments, or high-altitude settings—where the model’s stability and detection boundaries require further validation. Finally, implementation challenges arise when deploying the model on resource-constrained UAV platforms, including maintaining fast inference speeds, managing energy consumption, and ensuring seamless integration with existing hardware. Therefore, techniques such as ONNX/TensorRT optimization, model quantization, pruning, and the design of efficient data processing pipelines are critical for maintaining reliable real-time detection under such constraints.

## Conclusion

In this paper, we introduced SRD-YOLOv5, an enhanced YOLOv5n-based framework for UAV remote sensing object detection. By integrating the Scale Sequence Feature Fusion Module (SSFF), Multi-Scale Feature Extraction Module (MSFE), and an Extremely Small Target Detection Layer (ESTDL), our approach achieves significantly higher detection accuracy for small and densely distributed targets while maintaining low computational overhead. The Decoupled Head design further improves localization performance by independently optimizing regression and classification tasks. Experimental evaluations on challenging UAV datasets demonstrate that SRD-YOLOv5 outperforms existing methods in both accuracy and efficiency, making it well-suited for real-time applications under resource-constrained conditions.

Building on these findings, future work will focus on handling complex environmental factors—such as extreme weather and low-light scenarios—and exploring advanced architectures (e.g., attention mechanisms and transformers) to further enhance detection robustness and scalability.
